# Relaxation of selective constraints shapes variation of toll-like receptors in a colonial waterbird, the black-headed gull

**DOI:** 10.1007/s00251-020-01156-8

**Published:** 2020-01-30

**Authors:** Patrycja Podlaszczuk, Piotr Indykiewicz, Janusz Markowski, Piotr Minias

**Affiliations:** 1 Department of Biodiversity Studies and Bioeducation, Faculty of Biology and Environmental Protection, University of Łódź, Banacha 1/3, 90-237 Łódź, Poland; 2 Department of Biology and Animal Environment, Faculty of Animal Breeding and Biology, UTP University of Science and Technology, Mazowiecka 28, 85-084 Bydgoszcz, Poland

**Keywords:** dN/dS, Migration, Pathogen-driven selection, Selective constraint, Sociality

## Abstract

Nonspecific innate immune response is activated by toll-like receptors (TLRs), which recognize conserved molecular motifs characteristic for a broad spectrum of pathogens. In this study, we examined nucleotide substitution patterns and allelic diversity at five TLR genes in a wild nonpasserine bird, the black-headed gull *Chroicocephalus ridibundus*. We hypothesized that balancing selection can maintain high allelic diversity of TLR genes in the black-headed gull because of its ecological characteristics, coloniality, and migratoriness, which are associated with increased exposure and transmission of pathogens. Although we found moderately high levels of sequence polymorphism (8–49 haplotypes retrieved per locus within a sample of 60 individuals), most of these haplotypes were recorded at low frequencies within our study population. At the same time, we found no convincing evidence for the role of balancing selection in the maintenance of this variation (Tajima’s D < 0.5), and sites with a significant excess of nonsynonymous mutations (dN/dS > 1) were recorded only at two loci (TLR5 and TLR7). This pattern is consistent with relaxation of selective constraints, where most mutations are slightly deleterious and usually removed by purifying selection. No differences in the diversity and nucleotide substitution rates were found between endosomal loci responsible for viral RNA sensing and loci responsible for the recognition of extracellular pathogens. Our study provides the first information on evolutionary mechanisms shaping polymorphism of TLRs in a species from Lari suborder (gulls and allies) and suggests that TLR genes may be poorly responsive to ecological and life-history characteristics of hosts.

## Introduction

Hosts can cope with infection using two types of immune responses: innate and acquired. Acquired immune response is associated with an activity of the major histocompatibility complex (MHC). The main function of MHC molecules is to bind antigens derived from pathogens and display them on the cell surface for recognition by the appropriate T-cells (Janeway et al. 2005). In contrast, nonspecific innate immune response is activated by toll-like receptors (TLRs), which recognize conserved antigens characteristic for a broad spectrum of pathogens (so called pathogen-associated molecular patterns, PAMPs) (Medzhitov 2001).

Pathogen-driven selective pressure can considerably differ between species with different ecological and life-history characteristics. So far, evolutionary associations between bird ecology and the strength of pathogen-driven selection have been most extensively studied for MHC genes. A recent work by Minias et al. (2017) identified coloniality and migratoriness as the major determinants of selection patterns at the MHC. In colonial birds, pathogen-driven selection might be higher due to the strong horizontal transmission of pathogens (transmission of infection between individuals that are not in a parent-child relationship). Horizontal parasite transmission is usually positively density-dependent (McCallum et al. 2001), and colonial breeding is associated with high local host density, resulting in the higher prevalence and richness of pathogens and parasites (Côté and Poulin 1995). Similarly, long-distance migrants might be exposed to more diverse pathogen faunas throughout their annual cycle, resulting in stronger selective pressure on immune receptors when compared to resident species (Minias et al. 2017). Finally, it has been shown that pathogen-driven selection patterns can vary between genes responsible for recognition of different types of pathogens. For example, nonpasserines showed stronger selection at MHC class II, which is primarily involved in the recognition of extracellular pathogens, while passerines showed stronger selection at MHC class I (Minias et al. 2018), which is involved in the recognition of intracellular pathogens (but see Joffre et al. (2012) for antigen cross-presentation mechanisms). These contrasting patterns were primarily explained with various exposure of passerine and nonpasserine birds to different groups of pathogens, which may reflect general differences in body size and habitat between these two avian clades (Minias et al. 2018). Specifically, larger nonpasserine birds may be able to harbor more extracellular (but not necessarily intracellular) pathogens and parasites (Poulin 1995; Gregory et al. 1996; Morand and Poulin 2000; Arriero and Møller 2008), as well as they are more often associated with aquatic environments where the fauna of extracellular pathogens may be more diverse than in typical terrestrial habitats (e.g., Bush et al. 1990). It remains to be tested whether similar patterns hold true for TLRs.

TLRs are strictly conserved in their general structure, but an increasing body of evidence has supported the idea that different domains of TLR molecules likely evolve under different selection regimes. There are two major types of selection acting on immune receptors: purifying selection which acts to remove nonsynonymous (amino acid altering) mutations from gene sequence (depletes genetic variation in a population) and balancing selection, which generates and maintains genetic variation in a population via a wide spectrum of evolutionary mechanisms, including negative frequency-dependent, overdominant, and fluctuating selection (Hughes and Nei 1988; Takahata and Nei 1990; Hedrick 2002). Although purifying selection is thought to be a dominant force in the evolutionary history of TLRs, some studies on TLR evolution in birds found compelling evidence for balancing selection acting on certain TLR residues. For example, comprehensive work by Grueber et al. (2014) provided evidence that balancing selection played an important role in the evolution of most avian TLRs, consistent with the role of these loci in pathogen recognition and the mechanism of host-pathogen coevolution. In birds, TLRs and their corresponding genes are particularly well characterized in the domestic chicken *Gallus gallus domesticus*, and the analysis by Vinkler et al. (2014) detected signatures of balancing selection within the Galloanserae lineage, where greater excess of nonsynonymous mutations was found at the ligand-binding regions (leucine-rich repeat motifs) of TLR4 and TLR5 compared to TLR7. A recent study by Velová et al. (2018) showed adaptive evolution in all members of the avian TLR protein family across high number of species and most avian orders. They also found that balancing selection acted more on the cell surface TLR loci responsible for recognition of extracellular pathogens (TLR1LA/B, TLR2A/B, TLR4, and TLR5) than on the endosomal TLRs responsible for recognition of intracellular pathogens (TLR3, TLR7, and TLR21). Despite the growing body of research on the mechanisms of TLR evolution in birds, information on the associations between TLR diversity and selection signatures with ecological traits are virtually lacking for wild avian populations (Králová et al. 2018).

Our aim was to study allelic diversity and selection on TLR genes in a nonpasserine bird, the black-headed gull *Chroicocephalus ridibundus*. First, we hypothesized that strong balancing selection should act on TLR genes in the black-headed gull due to its ecological characteristics. Black-headed gull is highly colonial and shows large degree of sociality outside the breeding season (Snow and Perrins 1998), which can increase horizontal transmission of pathogens. Also, birds from Central European populations migrate for winter to Western Europe and Mediterranean region (Snow and Perrins 1998) and, thus, can be exposed to diverse faunas of pathogens. Our second hypothesis was that allelic diversity and signature of pathogen-driven selection should be stronger at TLR genes responsible for the recognition of extracellular rather than intracellular pathogens, consistently with the recent findings on the evolutionary mechanism at avian TLRs (Velová et al. 2018) and MHC (Minias et al. 2018).

## Materials and methods

### General field procedures

The study was conducted in the black-headed gull colony near Kusowo village (53°15’N, 18°02’E) in northern Poland, in 2016. Gulls nested on a lake surrounded by an agricultural landscape. The size of the colony was estimated at 800 breeding pairs and nesting densities ranged from 2.23 pairs/10m^2^ in peripheries to 5.30 pairs/10m^2^ in the colony center. Adult gulls (*n* = 60) were captured across the entire colony between 13 and 30 April. All birds were captured during incubation using spring traps (Ecotone, Sopot, Polska), and trapping procedures did not cause losses in broods. Each bird was ringed, and ca. 20 μl of blood from the ulnar vein was collected onto FTA ClassicCards (Whatman, Maidstone, UK). All cards were dried and stored at room temperature until analysis, following recommendation by Gutiérrez-Corchero et al. (2002).

### DNA extraction, amplification, and sequencing

Nuclear DNA was extracted from blood stored on FTA cards using Bio-Trace DNA Purification Kit (EURx, Gdansk, Poland). A piece of dried blood sample (approx. 2 mm^2^) was cut away from each card with a sterile cutter and used for DNA extraction, which followed a manufacturer’s protocol (EURx, Gdansk, Poland). For PCR amplifications of TLR genes, we used conserved primers developed by Alcaide and Edwards (2011). All PCR amplifications were conducted in a final volume of 20 μl containing 10 μl of DreamTaq PCR Master Mix (Thermo Fisher Scientific Inc., Waltham, MA, USA) and 0.2 μM of each primer. One μl DNA extract from blood samples was added to each reaction. Annealing temperatures and PCR conditions followed the protocol of Alcaide and Edwards (2011). All PCR amplifications were checked on 2% agarose gels.

We obtained successful amplifications for five out of ten tested TLR loci (Table 1), including two endosomal loci (TLR3 and TLR7) responsible for single- and double-stranded viral RNA sensing and three loci responsible for the recognition of extracellular pathogens via sensing of diacylated lipoproteins from the cell wall of bacteria, fungi, and parasites (TLR1LB), lipopolysaccharides (LPS) from the cell wall of gram-negative bacteria (TLR4), and flagellins of the flagellated bacteria (TLR5) (Alcaide and Edwards 2011). These five loci were successfully amplified in all sampled birds (*n* = 60), and PCR products were sequenced with Sanger sequencing technology in both forward and reverse directions. All sequences were assembled, trimmed to uniform lengths within each locus, and aligned in Geneious 10.0.5 software (Biomatters Ltd., Auckland, New Zealand). Final size of each alignment ranged from 762 to 1203 bp (Table 1). Although we, on average, sequenced only 40% of the total coding region of each gene (1959–3180 bp according to Temperley et al. (2008)), we mostly focused on the sequencing of extracellular leucine-rich repeat motifs (LRRs) that are responsible for PAMP recognition. In fact, 71% of codons from our alignments were identified as forming LRRs, based on the chicken TLR structure described by Temperley et al. (2008). The most evolutionarily conserved TLR motifs, e.g., the cytoplasmic toll/interleukin 1 resistance (TIR) domain (Beutler and Rehli 2002), were not targeted by the primers. Also, no intron fragments were genotyped. Taking all this into account, TLR regions targeted at each locus are likely to provide a good representation of genetic variation associated with PAMP-recognition functions of each TLR protein.

**Table 1:** Polymorphism statistics for five TLR loci in the black-headed gull

Locus	Fragment size (bp)	No. of inferred haplotypes	No. of polymorphic sites	Average no. of nucleotide differences	Nucleotide diversity	Synonymous nucleotide diversity	Nonsynonymous nucleotide diversity
TLR1LB	1044	26	19	2.954	0.00283	0.00264	0.00019
TLR3	1128	16	11	1.927	0.00171	0.00159	0.00012
TLR4	831	8	7	0.448	0.00054	0.00040	0.00014
TLR5	1203	38	14	3.219	0.00268	0.00082	0.00186
TLR7	762	49	16	3.3	0.00433	0.00325	0.00108

Sequences from each locus were assigned to haplotypes using the PHASE algorithm (Stephens and Donelly 2003) in DnaSP v6.10.03 (Rozas et al. 2017). For this purpose, we used a burn-in of 1000 iterations, followed by 1000 iterations and a thinning interval of 10. The output probability threshold for haplotypes was set to 0.95, and haplotype inference was successful for all individuals (*n* = 60). All haplotypes were assembled and aligned in Geneious 10.0.5 software (Biomatters Ltd., Auckland New Zealand). All unique sequences were deposited in Genbank (accession nos: MN995080-MN995216).

### Phylogenetic analysis

To assess phylogenetic relationships of TLR sequences of the black-headed gull and other avian species, we searched for GenBank sequences showing highest pairwise identity with consensus black-headed gull sequences. For this purpose, we used BLAST search as implemented in Geneious software and extracted ten sequences per each TLR locus (one sequence extracted per species). All extracted sequences showed >90% pairwise identity with the query sequences of the black-headed gull. We also extracted TLR sequences from the common ostrich *Struthio camelus* (one sequence per locus) and used them as outgroups. Phylogenetic relationships were inferred using approximately maximum-likelihood approach, as implemented in FastTree v2.1.5 software (Price et al. 2010), which produces topologies of similar accuracy to the ones produced with standard maximum-likelihood methods (Liu et al. 2011). The trees were initially constructed with neighbor-joining method, and their topology was then refined with subtree-pruning-regrafting (SPRs), minimum-evolution nearest-neighbor interchanges (NNIs), and maximum-likelihood NNIs. General time-reversible (GTR) model of nucleotide substitution with a discrete Gamma distribution was used to account for different rates of evolution at different sites and for uncertainty in these rates (Yang 1994). Local support values were computed based on the Shimodaira-Hasegawa test (Shimodaira and Hasegawa 1999). Topologies were constructed using consensus black-headed gull sequences from each locus.

### Analysis of diversity and selection

Sequence polymorphism at each locus was measured as the number of segregating sites, total number of mutations, average number of nucleotide differences, and average nucleotide diversity using DnaSP software. In order to infer selection acting on TLR loci, we used three different approaches. First, signature of selection was tested with three population genetic tests based on the allele frequency spectrum: Fu and Li’s D* and F* (Fu and Li 1993), as well as Tajima’s D (Tajima 1989). Fu and Li’s tests are directly based on the coalescent, and they focus on the presence of singletons (i.e., polymorphisms that affect single individuals within a sample), whereas Tajima’s test compares the number of nucleotide differences between pairs of sequences and the number of polymorphic sites. All these tests indicate an excess of low frequency polymorphisms (negative values) or deficit of both low and high frequency polymorphisms (positive values), relative to the neutral expectation (zero value). The first scenario indicates population size expansion or negative/purifying selection, while the second is consistent with recent population bottleneck or positive/balancing selection (Bollmer et al. 2011). Although these tests may not clearly distinguish between demographic and selection alternatives, there is no evidence for recent demographic changes in our study species (relatively stable population size of ca. 100,000 breeding pairs in Poland), and, thus, we interpreted the results in terms of selection processes. Tajima’s D was not only calculated across all polymorphic sites at each locus but also separately for synonymous and nonsynonymous sites (D_syn_ and D_non_, respectively). Low D_non_ and high D_syn_ values indicate that rare variants are particularly common at nonsynonymous sites. This scenario is consistent with relaxation of selective constraint, where most mutations are slightly deleterious and usually removed by purifying selection (Hughes 2005). All summary statistics (D*, T*, and D) were estimated for each locus and tested for a departure from neutrality using DnaSP software.

Second, we assessed relative rates of nonsynonymous and synonymous nucleotide substitutions. Under negative or purifying selection, nonsynonymous substitutions accumulate more slowly than synonymous substitutions, and under positive or balancing selection, the opposite pattern occurs. Thus, the relative rate of nonsynonymous substitutions per nonsynonymous site (dN) to synonymous substitutions per synonymous site (dS) is acknowledged as a standard measure of selection over evolutionary time, where dN/dS < 1 indicates negative selection and dN/dS > 1 as positive selection (Nei and Gojobori 1986; Yang et al. 2000). Despite this expectation, dN/dS ratio may be insensitive to selection coefficient when sequences are sampled from a single population, as the differences between sequences may represent segregating polymorphisms rather than fixation events along independent lineages (Kryazhimskiy and Plotkin 2008). Under such circumstances, high dN/dS values may not necessarily indicate positive or balancing selection but can be indicative for the relaxation of selective constraint and the prevalence of slightly deleterious mutations (Adachi and Hasegawa 1996; Hasegawa et al. 1998; Kryazhimskiy and Plotkin 2008). The dN/dS ratio has commonly been used to infer molecular processes shaping variation of TLRs at the intraspecific level (Alcaide and Edwards 2011; Gonzalez-Quevedo et al. 2015; Nelson-Flower et al. 2018), although their interpretation in terms of selection may not be justified in this context. Because our sample originated from a single population, we interpreted high dN/dS as an indication for the relaxation of selective constraint, rather than as an evidence for balancing or positive selection acting on our target genes, which follows argumentation by Kryazhimskiy and Plotkin (2008).

Nucleotide substitution rates were estimated with both Bayesian and maximum-likelihood approaches implemented in the Datamonkey web server (Delport et al. 2010) for HyPhy package (Kosakovsky Pond et al. 2005) using three different methods: Fast Unconstrained Bayesian AppRoximation (FUBAR), Fixed Effects Likelihood (FEL), and Random Effects Likelihood (REL) (Kosakovsky Pond and Frost 2005; Murrell et al. 2013). FUBAR uses a Markov chain Monte Carlo (MCMC) routine that ensures a robustness against model misspecifications and leaves the distribution of selection parameters essentially unconstrained (Murrell et al. 2013). The other two methods (FEL and REL) are based on the maximum-likelihood estimates of the rate parameters, and under most scenarios, REL can suffer from higher rates of false positives than FEL approach, which is more conservative in terms of type I error (Kosakovsky Pond and Frost 2005). All analyses were run with default settings and input trees built from the sequence alignments. Amino acid residues with posterior probabilities >0.95 (FUBAR) or *p* < 0.05 (FEL and REL) were considered to have enough support for a significant excess of nonsynonymous or synonymous nucleotide substitutions. All these analyses were rerun for the set of multispecies TLR sequences extracted for phylogenetic reconstruction. This was used to compare positions of codons with significant excess of nonsynonymous substitutions identified in our study of black-headed gull population with those identified at the interspecific level.

Third, we compared the relative fit of different codon-based models of sequence evolution using CodeML PAML software (Yang 2007). We fitted three basic models: (*i*) M0, which assumes a single nucleotide substitution rate across all sites; (*ii*) M7, which assumes nearly neutral nucleotide substitution rates (0 < dN/dS < 1) with variation that follows β distribution; and (*iii*) M8, which assumes that proportion of sites may show an excess of nonsynonymous substitutions (dN/dS > 1) and variation in nucleotide substitution rates follows β distribution. The best-fitting models were selected using the Akaike Information Criterion (ΔAIC) (Posada and Buckley 2004), and sites with an excess of nonsynonymous substitutions under the M8 model were identified with Bayes empirical Bayes (BEB) algorithm (Yang et al. 2005).

Nucleotide substitution rates reported in the text were estimated using FUBAR approach. In this method, inference of dN and dS values is governed by the instantaneous rate matrix that also includes nucleotide mutational biases and equilibrium frequency parameters (Murrell et al. 2013). Then, probability of codon change at each site along every branch is recorded using the branch length parameter in the corresponding element of the transition matrix (Murrell et al. 2013). Consequently, reported dN and dS estimates do not represent the raw number of nonsynonymous/synonymous differences per nonsynonymous/synonymous site.

## Results

**Fig. 1:**
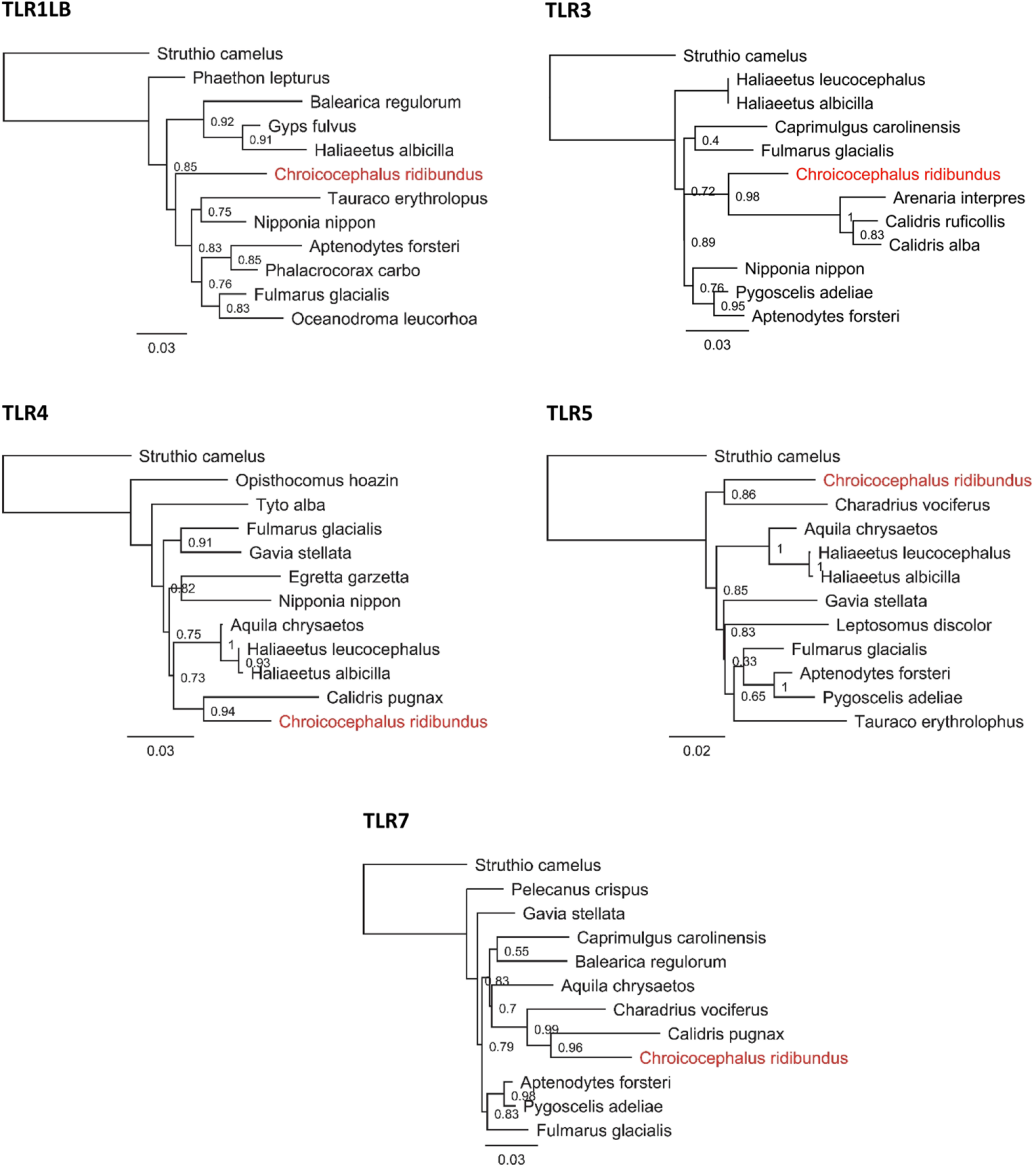
Consensus maximum-likelihood topology for five TLR loci in the black-headed gull *Chroicocephalus ridibundus* (marked in red) and ten other bird species showing highest pairwise identity of sequences (as retrieved from GenBank). Local bootstrap support is shown at the nodes and scale bards indicate genetic distance in units of nucleotide substitutions per site. The common ostrich *Struthio camelus* was used as the outgroup

TLR sequences retrieved from the black-headed gull clustered with sequences of closely related Charadriiform species (Charadrii and Scolopaci suborders), if they were available (Fig. 1). Node support for these clusters was high (0.86–0.96; Fig. 1). No Charadriiform sequences were available for TLR1LB, and black-headed gull sequences from this locus did not show any close phylogenetic relationships with sequences of other bird species.

In the black-headed gull, the number of TLR haplotypes ranged from 8 to 49 per locus, and the number of polymorphic sites was between 7 and 19 (Table 1). TLR7 was the most polymorphic among all the loci in terms of haplotype number and the number of polymorphic sites, when variation in fragment size was taken into account (2.10 polymorphic sites per 100 bp). TLR7 also showed highest average number of nucleotide differences and nucleotide diversity (Table 1). TLR4 was recognized as the least polymorphic locus (0.84 polymorphic sites per 100 bp). We found no differences in polymorphism between endosomal RNA-sensing TLRs (TLR3 and TLR7) and other loci (1.28 ± 0.29 vs. 1.54 ± 0.56 polymorphic sites per 100 bp, t test: *p* = 0.67; 2.21 ± 0.88 vs. 2.61 ± 0.69 nucleotide differences, t test: *p* = 0.77; 0.0020 ± 0.0007 vs. 0.0030 ± 0.0013 nucleotide diversity, t test: *p* = 0.52).

Frequencies of most haplotypes retrieved from our study population were generally low, and 67.2% of all haplotypes (across all loci examined) had extremely low frequencies (<2%). All TLR loci except for TLR4 showed two or three haplotypes with frequencies >10%. TLR4 showed a single dominant haplotype (80.8% frequency), and all other haplotypes had frequencies <10%. Three loci (TLR1LB, TLR3, and TLR4) showed a single dominant amino acid variant (88.3–94.2% frequencies), while TLR5 and TLR7 loci showed two and three amino acid variants with >10% frequencies, respectively.

**Fig. 2:**
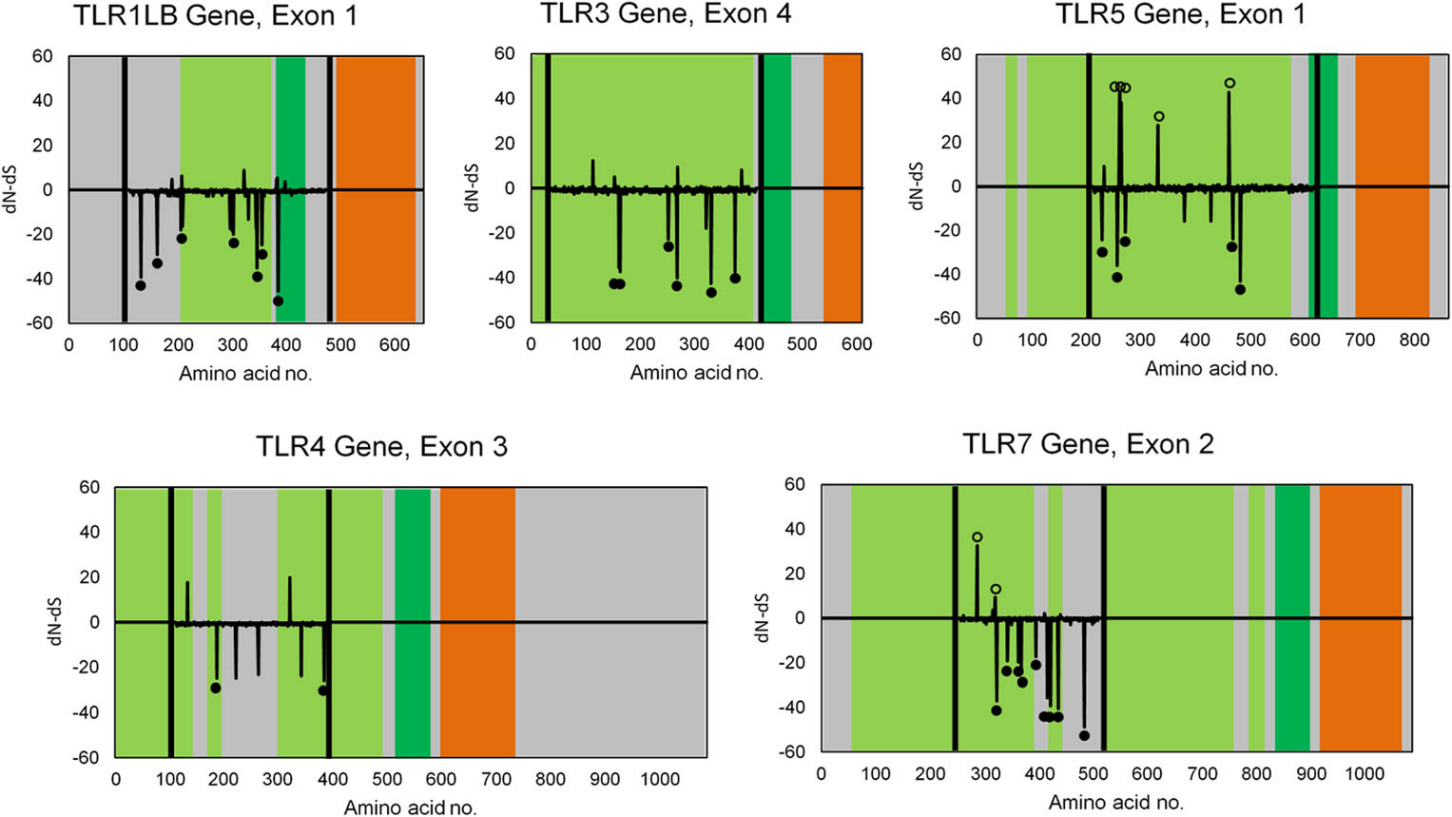
Nucleotide substitution rate (dN/dS) along five TLR loci in the black-headed gull. Black vertical lines mark exon regions targeted in this study. Open and full circles indicate amino acid residues with a significant excess of nonsynonymous and synonymous nucleotide substitutions, respectively, as inferred with FUBAR analysis. Colors represent different TLR domains: light green – LRR domains, dark green – C-terminal LRR domains, orange – TIR signaling domains, gray – coding regions with no specific function recognized. TLR structure was based on the updated annotations of chicken protein sequences provided by Temperley et al. (2008)

The dN/dS ratio was <1 for all loci (0.42–0.75; Table 2), and the number of sites with a significant excess of synonymous nucleotide substitutions (as identified by at least one analysis method) ranged from 4 to 13 per locus (Table 3). The mean dN/dS ratio for these sites was 0.059 ± 0.006 (0.050–0.125 per locus). The number of sites with significant excess of synonymous nucleotide substitutions, as identified by at least two analysis methods, was slightly lower and ranged from one to eight (Table 3). Interestingly, sites with a significant excess of nonsynonymous nucleotide substitutions were recorded only at two loci: TLR5 (five sites) and TLR7 (two sites) (Table 2; Fig. 2). The mean dN/dS ratio for these sites was 15.8 ± 2.7. Only three sites (two at TLR5 and one at TLR7) were identified as showing a significant excess of nonsynonymous nucleotide substitutions by at least two analysis methods (Table 3). The presence of sites with an excess of nonsynonymous mutations at TLR5 was confirmed with comparisons of different codon-based models of sequence evolution. The model allowing for the presence of sites with dN/dS > 1 (M8) was identified as best-fitting to the pattern of nucleotide substitutions at this locus (Table 4). The analysis identified five codons with significant excess of nonsynonymous substitutions (average dN/dS = 8.63, posterior probability >0.95), and location of these sites was consistent with the results of FUBAR analysis (Tables 3, and 4). One site showing a significant excess of nonsynonymous nucleotide substitutions in the black-headed gull (position 48, TLR5) was identified as being under balancing/positive selection (dN/dS = 5.77) in the interspecific analysis. There was no difference in the dN/dS ratio between endosomal RNA-sensing TLRs and other loci in the black-headed gull (0.74 ± 0.05 vs. 0.81 ± 0.12, t test: *p* = 0.56).

**Table 2:** The number of amino acid sites with significant excess of nonsynonymous/synonymous substitutions (combined results of FUBAR, REL, and FEL) and estimates of nucleotide substitution rates (FUBAR) at five TLR loci in the black-headed gull

Locus	No. of sites with an excess of nonsynonymous substitutions	No. of sites with an excess of synonymous substitutions	dN	dS	dN/dS
TLR1LB	0	13	1.29	2.87	0.45
TLR3	0	7	1.75	3.26	0.54
TLR4	0	4	3.53	4.69	0.75
TLR5	5	7	1.72	2.68	0.64
TLR7	2	10	0.98	2.33	0.42

**Table 3:** Location of amino acid sites with significant excess of synonymous (dN/dS < 1) and nonsynonymous (dN/dS > 1) nucleotide substitutions at five TLR loci in the black-headed gull, as inferred with FEL, REL, and FUBAR methods

Locus	Excess of nucleotide substitutions	Site	dN/dS FUBAR	dN/dS REL	dN/dS FEL
TLR1LB	Synonymous	21	0.030	0.004	0.000
		51	0.057	0.010	–
		95	–	0.011	–
		98	–	0.011	–
		185	–	0.011	–
		189	–	0.011	–
		191	0.049	0.010	0.000
		219	–	0.012	–
		233	–	0.011	–
		235	0.030	0.006	0.000
		244	0.065	0.011	–
		273	0.065	0.010	–
		274	0.022	0.003	0.000
TLR3	Synonymous	124	0.051	0.003	0.000
		126	0.075	0.003	–
		215	0.053	0.003	–
		231	0.045	0.003	0.000
		285	–	0.003	–
		294	0.026	0.003	0.000
		338	0.049	0.003	0.000
TLR4	Synonymous	76	0.121	–	0.000
		111	–	–	0.000
		231	–	–	0.000
		273	0.104	–	–
TLR5	Nonsynonymous	48	9.037	–	–
		49	15.634	–	1.62E+16
		51	11.332	–	–
		118	18.102	225.250	–
		248	13.862	–	–
	Synonymous	16	0.050	0.003	–
		44	0.038	0.001	0.000
		59	0.034	0.001	0.000
		167	–	0.002	–
		215	–	0.003	–
		255	0.032	0.001	0.000
		269	0.068	0.004	0.000
TLR7	Nonsynonymous	31	30.808	280.034	1.55E+15
		64	11.962	–	–
	Synonymous	67	0.033	0.003	0.000
		87	0.031	0.005	0.000
		107	0.030	0.005	0.000
		112	0.051	0.003	0.000
		139	0.038	0.006	0.000
		160	0.015	0.002	0.000
		166	0.026	0.002	0.000
		180	0.031	0.003	0.000
		228	0.013	0.001	0.000
		243	–	0.009	–

FEL assumes zero nonsynonymous substitution rate at sites where no nonsynonymous substitutions were observed, resulting in zero dN/dS ratios. High dN/dS ratios for sites with an excess of nonsynonymous rates (especially under FEL and REL methods) are due to very low dS values.

**Table 4:** Relative fit of different codon-based models of sequence evolution, as assessed with CodeML PAML software

Locus	Model	ln L	AIC	ΔAIC	Sites with an excess of nonsynonymous substitutions
TLR1LB	M0	−1653.59	3309.18	0.00	–
	M7	−1653.59	3311.18	2.00	–
	M8	−1666.18	3340.36	31.18	–
TLR3	M0	−1713.47	3428.94	1.02	–
	M7	−1711.96	3427.92	0.00	–
	M8	−1711.00	3430.00	2.08	–
TLR4	M0	−1193.97	2389.94	0.00	–
	M7	−1193.97	2391.94	2.00	–
	M8	−1194.00	2396.00	6.06	–
TLR5	M0	−2022.75	4047.50	59.20	–
	M7	−2001.66	4007.32	19.02	–
	M8	−1990.15	3988.30	0.00	48, 49, 51, 118, 248
TLR7	M0	−1484.16	2970.32	14.22	–
	M7	−1476.05	2956.10	0.00	–
	M8	−1491.40	2990.80	34.70	–

Best-fitting models are marked in bold.

Fu and Li’s D* and F* tests provided no support for significant deviation from neutrality at any of five TLR loci (all *p* > 0.1), although TLR5 showed markedly higher (positive) D* and F* values than other loci (Table 5). TLR5 and TLR7 were the only loci with positive Tajima’s D (Table 2), although none of the values differed significantly from zero (all p > 0.1). Tajima’s D estimates for nonsynonymous sites (D_non_) were negative for all loci examined except for TLR5, although departures of D_non_ from zero were significant or approached significance (*p* < 0.1) only for TLR1LB and TLR3. TLR1LB, TLR3, and TLR7 had negative D_non_ and positive D_syn_ values, indicating that rare variants at these loci were particularly common at nonsynonymous sites and being consistent with relaxation of selective constraints. TLR4 had negative D_non_ and D_syn_ values (Table 5), indicating that rare variants were common at both nonsynonymous and synonymous sites, which may indicate a signature of purifying selection. This pattern was consistent with the lowest Tajima’s D value recorder across all sites at TLR4 (D = −1.50, Table 5). TLR5 was the only locus with positive D_non_ and negative D_syn_ values, suggesting a possible signature of balancing selection.

**Table 5:** The results of site and allele frequency spectrum tests: Fu and Li’s D* and F* and Tajima’s D

Locus	D*	F*	Tajima’s D		
			All polymorphic sites (D)	Synonymous sites (D_syn_)	Nonsynonymous sites (D_non_)
TLR1LB	0.76	0.35	−0.47	0.36	−1.80**
TLR3	0.05	−0.03	−0.16	0.85	−1.58*
TLR4	0.28	−0.38	−1.50	−1.32	−1.04
TLR5	1.02	0.94	0.41	−0.73	1.62
TLR7	−0.52	−0.27	0.29	0.82	−0.58

Statistical significance estimates is marked as following: * 0.10 < P < 0.05, ** P < 0.05

## Discussion

Our study revealed that TLR genes in the black-headed gull showed moderately high levels of sequence polymorphism, but this genetic diversity was most likely generated via the relaxation of selective constraints, as we found no convincing evidence for balancing selection acting across five TLR loci. We found no differences in polymorphism or selection between endosomal RNA-sensing TLRs and other loci responsible for the recognition of extracellular pathogens. TLR5 and TLR7, which recognize bacterial flagellin and endosomal viral RNA, respectively, were recognized as the most polymorphic among five TLR loci included in our study, but only TLR5 showed some signature of balancing selection, as indicated by high (although nonsignificant) Tajima’s D value across nonsynonymous sites and the best fit of sequence evolution model (M8) that allows for the presence of sites with an excess of nonsynonymous substitutions.

The signature of pathogen-driven balancing selection at the five TLR genes examined was negligible in the black-headed gull, despite life-history characteristics which are likely to be associated with increased pathogen exposure: coloniality and migratory behavior. Thus, our results suggest that ecological factors associated with high pathogen exposure may not exert strong influence on the allelic diversification of TLR genes in our study gull population. This stays in stark contrast to the previous research on the MHC genes, which code for pathogen-recognition receptors of the acquired part of the immune system. For example, recent work by Minias et al. (2017) provided evidence that the strength of balancing selection on MHC genes in birds was strongly related to their social and migratory behavior. Migratory and colonial species experienced stronger balancing selection on the MHC than resident and solitary species (dN/dS = 4.20 ± 0.95 vs. dN/dS = 1.92 ± 0.26 for migratory and resident species, respectively; dN/dS = 4.05 ± 0.72 vs. dN/dS = 2.50 ± 0.34 for colonial and solitary species, respectively) (Minias et al. 2017). This pattern was explained by greater exposure of migratory species to more diverse pathogen and parasite faunas throughout their annual cycle and increased horizontal transmission of pathogens in colonial species.

So far, TLR diversity and structure were well characterized in a limited number of model species, including domestic chicken as a universal avian model. In contrast, mechanisms shaping polymorphism at TLRs have rarely been assessed in wild avian populations. To our knowledge, this study is one of the few that assessed diversity and nucleotide substitution rates at TLRs in nonpasserine birds. Most data on TLR diversity in wild birds originates from the studies of passerines, which show contrasting life histories to our study species (mostly solitary and short-distant migrants). For example, recent work of Nelson-Flower et al. (2018) found no evidence for codon-specific episodic positive selection, but detected purifying (negative) selection acting at four TLR loci (TLR1LB, TLR3, TLR4, TLR15) in the song sparrow *Melospiza melodia*, a short-distance migrant with a broad breeding range across North America. Surprisingly, much higher TLR diversity was recorded in a resident passerine, the house finch *Carpodacus mexicanus* (Alcaide and Edwards 2011), which showed up to 62 haplotypes per locus in a sample ca. 50 individuals (Alcaide and Edwards 2011). At the same time, most TLR loci in house finch showed similar proportions of synonymous versus nonsynonymous substitutions (Alcaide and Edwards 2011), which is in stark contrast to our results on the black-headed gull.

Other research focused on examining TLR diversity within isolated or bottlenecked avian populations (Grueber et al. 2012, 2015; Gilroy et al. 2017a, b; Knafler et al. 2017). For example, TLR5 was one of the few that showed nonsynonymous polymorphisms and one of the three loci showing the greatest diversity in the Seychelles warbler *Acrocephalus sechellensis*, an island endemic passerine species (Gilroy et al. 2017a). Similarly, TLRs in a bottlenecked population of a New Zealand robin *Petroica australis rakiura* showed low level of variation (2–5 haplotypes per locus) (Grueber et al. 2012), while TLR variation in the endemic Berthelot’s pipit *Anthus berthelotii* was much reduced compared to the more widespread tawny pipit *Anthus campestris*, reflecting the bottleneck history of the first species (Gonzalez-Quevedo et al. 2015). Although this kind of research is crucial to understand molecular mechanism associated with island colonization events and may have important role in conservation of endangered species, low levels of TLR sequence polymorphism provide limited insight into the general patterns of pathogen-driven selection within wild bird populations.

Information on the mechanisms shaping TLR diversity in nonpasserine birds is even scarcer. Extracellular TLRs (TLR4 and TLR5) in the gray partridge *Perdix perdix* showed slightly higher diversity than the endosomal viral-sensing TLR locus (TLR7). All these loci appeared to be fully functional, but they showed generally low level of variation (less than five haplotypes per locus) due to either negative selection or a population bottleneck (Vinkler et al. 2015). In contrast, TLR1LA locus in white-winged flufftail *Sarothrura ayresi* was the only one which showed an excess of nonsynonymous to synonymous mutations, although no statistical support was found for balancing selection acting on any of its residues (Dalton et al. 2016). All other four loci (TLR1LB, TLR3, TLR4, and TLR7) either showed negligible variation or dN/dS < 0.5, indicating for the predominant role of purifying selection (Dalton et al. 2016). Similarly, TLR3 and TLR7 showed significantly negative Tajima’s D values in the mallard *Anas platyrhynchos*, which could be due to purifying selection (Jax 2019). No evidence for balancing selection was observed at any of TLR loci in this species (Jax 2019). In contrast, high TLR diversity was recorded in a colonial bird of prey, the common kestrel *Falco naumanni* (Alcaide and Edwards 2011). TLR5 was found to be most polymorphic among all loci examined (16 haplotypes detected in 8 individuals), and it was the only locus that showed marked excess of nonsynonymous versus synonymous nucleotide substitutions (Alcaide and Edwards 2011). This is consistent with our results for the colonial black-headed gull, in which TLR5 showed one of the highest polymorphism and had the greatest number of sites with a significant excess of nonsynonymous nucleotide substitutions. TLR5 was also the only locus with high positive value of Tajima’s D calculated for nonsynonymous sites (D_non_), which may be indicative for balancing selection. Finally, our comparisons of different codon-based models of sequence evolution identified TLR5 as the only locus, where the pattern of nucleotide substitutions was best explained by the model allowing for the presence of positive selection (dN/dS > 1). Our results strongly suggest that polymorphism at all the other TLR loci examined in our study was generated by the relaxation of selective constraints rather than via balancing selection. As far as we are aware, our study is the first to explicitly show the role of this mechanism in shaping genetic variation of avian TLRs. So far, selection patterns at TLRs within an order of Charadriiformes were studied only in three species of long-distance migratory waders, which showed predominance of purifying selection at the two RNA-sensing loci (TLR3 and TLR7) (Raven et al. 2017). Thus, our study is also the first to provide information on the polymorphism and nucleotide substitutions rates at TLRs in a species from Lari suborder (gulls, terns, skuas, skimmers, and auks).

Global analyses of substitution rates at avian TLRs showed large variation in the signatures of episodic diversifying selection between the loci. Pioneer research by Grueber et al. (2014) on data from up to 20 wild bird species per locus revealed the highest proportion on sites evolving under positive or balancing selection (>2%) at TLR3, TLR4, and TLR5. However, more recent analyses that were based on larger sample sizes (40–54 species per locus) found that TLR1LB, TLR2A, and TLR5 had >5% proportion of sites with a significant excess of nonsynonymous nucleotide substitutions (Velová et al. 2018). Our results for the black-headed gull only partially fit into these general patterns, as a possible signature of balancing selection was found only for TLR5. However, it must be acknowledged that conserved primers used in this study, originally developed by Alcaide and Edwards (2011), targeted only an average of 40% of the entire coding region at each locus, although our sequencing approach focused on the extracellular LRR motifs that recognize PAMPs. Also, our study was limited to five out of ten avian TLR loci. Thus, we recommend that future studies should aim for a more comprehensive assessment of TLR genes in larids, possibly taking an advantage of de novo genome assembly or RNAseq, which should enhance development of species-specific primers.

In conclusion, our results provided little evidence for balancing selection acting on five of the TLRs in the colonial migratory nonpasserine species, the black-headed gull. Instead, most polymorphism of TLRs observed in our study population was most likely slightly deleterious and generated through the relaxation of selective constrains. The TLR family of receptors has a highly conserved structural architecture, and strong selective pressure for the recognition of specific classes of PAMPs can maintain a largely homogeneous repertoire of TLR loci across species. Thus, it seems that highly variable MHC genes may be evolutionarily more responsive to ecological and life-history traits of hosts than TLRs, which usually retain their conserved structure across phylogenetically and ecologically divergent taxa.

## Data Availability

All sequences were deposited in GenBank (accession nos: MN995080-MN995216).

## References

[CR1] Adachi J, Hasegawa M (1996). Tempo and mode of synonymous substitutions in mitochondrial DNA in primates. Mol Biol Evol.

[CR2] Alcaide M, Edwards SV (2011). Molecular evolution of the toll-like receptor multigene family in birds. Mol Biol Evol.

[CR3] Arriero E, Møller AP (2008). Host ecology and life-history traits associated with blood parasite species richness in birds. J Evol Biol.

[CR4] Beutler B, Rehli M (2002). Evolution of the TIR, tolls and TLRs: functional inferences from computational biology. Curr Top Microbiol Immunol.

[CR5] Bollmer JL, Ruder EA, Johnson JA, Eimes JA, Dunn PO (2011). Drift and selection influence geographic variation at immune loci of prairie-chickens. Mol Ecol.

[CR6] Bush AO, Aho JM, Kenndy CR (1990). Ecological versus phylogenetic determinants of helminth parasite community richness. Evol Ecol.

[CR7] Côté IM, Poulin R (1995). Parasitism and group size in social animals: a meta-analysis. Behav Ecol.

[CR8] Dalton DL, Vermaak E, Smit-Robinson HA, Kotze A (2016). Lack of diversity at innate immunity toll-like receptor genes in the critically endangered white-winged flufftail (Sarothrura ayresi). Sci Rep.

[CR9] Delport W, Poon AF, Frost SDW, Kosakovsky Pond SL (2010). Datamonkey 2010: a suite of phylogenetic analysis tools for evolutionary biology. Bioinformatics.

[CR10] Fu YX, Li WH (1993). Statistical tests of neutrality of mutations. Genetics.

[CR11] Gilroy DL, Phillips KP, Richardson DS, Van Oosterhout C (2017). Toll-like receptor variation in the bottlenecked population of the Seychelles warbler: computer simulations see the ‘ghost of selection past’and quantify the ‘drift debt’. J Evol Biol.

[CR12] Gilroy DL, Van Oosterhout C, Komdeur J, Richardson DS (2017). Toll-like receptor variation in the bottlenecked population of the endangered Seychelles warbler. Anim Conserv.

[CR13] Gonzalez-Quevedo C, Spurgin LG, Illera JC, Richardson DS (2015). Drift, not selection, shapes toll-like receptor variation among oceanic island populations. Mol Ecol.

[CR14] Gregory RD, Keymer AE, Harvey PH (1996). Helminth parasite richness among vertebrates. Biodivers Conserv.

[CR15] Grueber CE, Wallis GP, King TM, Jamieson IG (2012). Variation at innate immunity toll-like receptor genes in a bottlenecked population of a New Zealand robin. PLoS One.

[CR16] Grueber CE, Wallis GP, Jamieson IG (2014). Episodic positive selection in the evolution of avian toll-like receptor innate immunity genes. PLoS One.

[CR17] Grueber CE, Knafler GJ, King TM, Senior AM, Grosser S, Robertson B, Weston KA, Brekke P, Harris CLW, Jamieson IG (2015). Toll-like receptor diversity in 10 threatened bird species: relationship with microsatellite heterozygosity. Conserv Genet.

[CR18] Gutiérrez-Corchero F, Arruga MV, Sanz L, Garcia C, Hernandez MA, Campos F (2002). Using FTA cards to store avian blood samples for genetic studies. Their application in sex determination. Mol Ecol Notes.

[CR19] Hasegawa M, Cao Y, Yang Z (1998). Preponderance of slightly deleterious polymorphisms in mitochondrial DNA: nonsynonymous/synonymous rate ratio is much higher within species than between species. Mol Biol Evol.

[CR20] Hedrick PW (2002). Pathogen resistance and genetic variation at MHC loci. Evolution.

[CR21] Hughes AL (2005). Evidence for abundant slightly deleterious polymorphisms in bacterial populations. Genetics.

[CR22] Hughes AL, Nei M (1988). Pattern of nucleotide substitution at major histocompatibility complex class I loci reveals overdominant selection. Nature.

[CR23] Janeway C, Travers P, Walport M, Shlomchick M (2005). Immunobiology: the immune system in health and disease.

[CR24] Jax E (2019). Immunology going wild: genetic variation and immunocompetence in the mallard (*Anas platyrhynchos*).

[CR25] Joffre OP, Segura E, Savina A, Amigorena S (2012). Cross-presentation by dendritic cells. Nat Rev Immunol.

[CR26] Knafler GJ, Grueber CE, Sutton JT, Jamieson IG (2017). Differential patterns of diversity at microsatellite, MHC, and TLR loci in bottlenecked. N Z J Ecol.

[CR27] Kosakovsky Pond SL, Frost SDW (2005). Not so different after all: a comparison of methods for detecting amino acid sites under selection. Mol Biol Evol.

[CR28] Kosakovsky Pond SL, Frost SDW, Muse SV (2005). HyPhy: hypothesis testing using phylogenies. Bioinformatics.

[CR29] Králová T, Albrecht T, Bryja J, Hořák D, Johnsen A, Lifjeld JT, Novotný M, Sedláček O, Velová H, Vinkler M (2018). Signatures of diversifying selection and convergence acting on passerine toll-like receptor 4 in an evolutionary context. Mol Ecol.

[CR30] Kryazhimskiy S, Plotkin JB (2008). The population genetics of dN/dS. PLoS Genet.

[CR31] Liu K, Linder R, Warnow T (2011). RAxML and FastTree: comparing two methods for large-scale maximum likelihood phylogeny estimation. PLoS One.

[CR32] McCallum H, Barlow N, Hone J (2001). How should pathogen transmission be modelled?. Trends Ecol Evol.

[CR33] Medzhitov R (2001). Toll-like receptors and innate immunity. Nat Rev Immunol.

[CR34] Minias P, Whittingham LA, Dunn PO (2017). Coloniality and migration are related to selection on MHC genes in birds. Evolution.

[CR35] Minias P, Pikus E, Whittingham LA, Dunn PO (2018). A global analysis of selection at the avian MHC. Evolution.

[CR36] Morand S, Poulin R (2000). Nematode parasite species richness and the evolution of spleen size in birds. Can J Zool.

[CR37] Murrell B, Moola S, Mabona A, Weighill T, Sheward D, Kosakovsky Pond SL, Scheffler K (2013). FUBAR: a fast unconstrained Bayesian AppRoximation for inferring selection. Mol Biol Evol.

[CR38] Nei M, Gojobori T (1986). Simple methods for estimating the numbers of synonymous and nonsynonymous nucleotide substitutions. Mol Biol Evol.

[CR39] Nelson-Flower MJ, Germain RR, MacDougall-Shackleton EA, Taylor SS, Arcese P (2018). Purifying selection in the toll-like receptors of song sparrows *Melospiza melodia*. J Hered.

[CR40] Posada D, Buckley TR (2004). Model selection and model averaging in phylogenetics: advantages of Akaike information criterion and Bayesian approaches over likelihood ratio tests. Syst Biol.

[CR41] Poulin R (1995). Phylogeny, ecology, and richness of parasite communities in vertebrates. Ecol Monogr.

[CR42] Price MN, Dehal PS, Arkin AP (2010). FastTree2 – approximately maximum-likelihood trees for large alignments. PLoS One.

[CR43] Raven N, Lisovski S, Klaassen M, Lo N, Madsen T, Ho SY, Ujvari B (2017). Purifying selection and concerted evolution of RNA-sensing toll-like receptors in migratory waders. Infect Genet Evol.

[CR44] Rozas J, Ferrer-Mata A, Sánchez-DelBarrio JC, Guirao-Rico S, Librado P, Ramos-Onsins SE, Sánchez-Gracia A (2017). DnaSP 6: DNA sequence polymorphism analysis of large data sets. Mol Biol Evol.

[CR45] Shimodaira H, Hasegawa M (1999). Multiple comparisons of log-likelihoods with applications to phylogenetic inference. Mol Biol Evol.

[CR46] Snow DW, Perrins CM (1998). The birds of the Western Palearctic.

[CR47] Stephens M, Donelly P (2003). A comparison of bayesian methods for haplotype reconstruction from population genotype data. Am J Hum Genet.

[CR48] Tajima F (1989). Statistical method for testing the neutral mutation hypothesis by DNA polymorphism. Genetics.

[CR49] Takahata N, Nei M (1990). Allelic genealogy under overdominant and frequency dependent selection and polymorphism of major histocompatibility complex loci. Genetics.

[CR50] Temperley ND, Berlin S, Paton IR, Griffin DK, Burt DW (2008). Evolution of the chicken toll-like receptor gene family: a story of gene gain and gene loss. BMC Genomics.

[CR51] Velová H, Gutowska-Ding MW, Burt DW, Vinkler M, Yeager M (2018). Toll-like receptor evolution in birds: gene duplication, pseudogenisation and diversifying selection. Mol Biol Evol.

[CR52] Vinkler M, Bainová H, Bryja J (2014). Protein evolution of toll-like receptors 4, 5 and 7 within Galloanserae birds. Genet Sel Evol.

[CR53] Vinkler M, Bainová H, Bryjová A, Tomášek O, Albrecht T, Bryja J (2015). Characterisation of toll-like receptors 4, 5 and 7 and their genetic variation in the grey partridge. Genetica.

[CR54] Yang Z (1994). Maximum likelihood phylogenetic estimation from DNA sequences with variable rates over sites: approximate methods. J Mol Evol.

[CR55] Yang Z (2007). PAML 4: phylogenetic analysis by maximum likelihood. Mol Biol Evol.

[CR56] Yang Z, Nielsen R, Goldman N, Pedersen AMK (2000). Codon-substitution models for heterogeneous selection pressures at amino acid sites. Genetics.

[CR57] Yang Z, Wong WS, Nielsen R (2005). Bayes empirical Bayes inference of amino acid sites under positive selection. Mol Biol Evol.

